# A bibliometric analysis of scientific literature in digital dentistry from low- and lower-middle income countries

**DOI:** 10.1038/s41405-024-00225-4

**Published:** 2024-05-25

**Authors:** Samira Adnan, Abhishek Lal, Nighat Naved, Fahad Umer

**Affiliations:** 1 Department of Operative Dentistry, Sindh Institute of Oral Health Sciences, Jinnah Sindh Medical University, Karachi, Pakistan; 2 Department of Medicine, The Aga Khan University, Karachi, Pakistan; 3 Section of Dentistry, Department of Surgery, The Aga Khan University, Karachi, Pakistan

**Keywords:** Dental education, Dental public health

## Abstract

**Objective:**

Bibliometric analysis and citation counts help to acknowledge influence of publications. The aim of this study was to conduct bibliometric and citation analysis of top-cited articles, from low- and lower-middle income countries, on use and application of digital technology in dentistry.

**Methodology:**

A search strategy based on “Digital Dentistry”, “Low Income Countries”, and “Lower-Middle Income Countries” was used in October 2023 using Scopus database to retrieve articles relevant to digital dentistry, with citation count of 10 or more. From 44 included articles, bibliometric information was analyzed on SPSS version 23. Network analysis based on co-citations, keywords, and number of citations was conducted on VOS software (version 1.6.20).

**Results:**

Most relevant articles were published in 2021 (*n* = 8), with 52.3% original articles, out of which 40.9% were in vitro studies. India had the highest number of articles (*n* = 24), with most publications in The Journal of Indian Prosthodontic Society (*n* = 4), and in the domain of General Dentistry (*n* = 15, 34.1%). Co-authorship network analysis was not significant, but country-wise co-authorship analysis revealed India with the greatest link strength (4.0). Highest occurring keyword was 3D printing (link strength 5.0), and the citation analysis revealed Journal of Prosthetic Dentistry with the most number of published documents (3), having a citation count of 275. Bibliographic coupling for sources revealed Journal of Indian Prosthodontic Society to have the highest link strength of 15.33.

**Conclusion:**

This analysis uncovers interesting bibliometric and citation based information including key thematic trends, emphasizing crucial role of technologies like 3D printing, CAD/CAM, and CBCT in digital dentistry. The study underscores the imperative for increased original research efforts in low- and lower middle-income countries.

## Introduction

Studies based on bibliometric data help to evaluate trends of scientific research that have been conducted in a particular domain, encompassing its significance and productivity over time. Based on various variables and indicators of impact, the included studies help identify relevant trends of focal interest. Researchers, institutes, journals, and specialties contributing and collaborating in the pertinent domain or for a core topic can be highlighted for interested researchers and the scientific community to follow, while simultaneously identifying gaps and avenues for further research. With the help of different software, network analysis of the articles could be carried out based on authors, keywords and similar parameters of interest, with identification of themes in the published literature on areas which researchers have focused on over the years, while simultaneously extrapolating what future research may be [1].

Citations received by scientific work are a key indicator of its impact [2]. Articles with a high number of citations can emerge as having the most influence on research and scientific work of other authors and provide findings that become the basis of extensive relevant exploration [3]. Despite their inherent limitations, citation counts can be used to acknowledge the influence of these publications [4]. Citation analysis has been conducted in various specialized domains of dentistry including regenerative endodontics [5], dental trauma [6, 7] and AI use in dentistry [8].

As with other medical fields, dentistry has been revolutionized by the introduction of digitalization in various aspects, and its utility and potential is still being explored. Different specialties of dentistry have benefited from the application of digital technology, in terms of efficiency and accuracy in diagnosis, treatment planning, procedures, data flow, patient records, management, and numerous other aspects [9]. The transformation which the use of digitalization has brought to imaging and scanning of the oral cavity, as well as assistance in designing and manufacturing of dental prostheses and other dental products is saving significant time and effort of dental specialists throughout the world [10]. The change from analog technology to digital technology might have been slow initially, but with the improved efficiency and various products that it offers, it is now considered a norm in clinical dental practice in developed countries.

Bibliometric analysis has been conducted on the use of digital technology in dentistry based on the number of citations [11, 12]. However, since most of the top-cited articles in majority of bibliometric studies hail from developed countries, there is a significant under-representation of scientific publications from low- and lower-middle income countries in this format of research, and digital dentistry is no exception. This common occurrence in most analysis of top-cited articles is probably because the scientific work from such regions is usually delayed in execution and completion when compared to resource-rich and higher-income countries, resulting from deficient funds and outdated technology needed to support relevant development. This delay leads to a lower citation count of published articles from low- and lower-middle income countries, since literature from developed countries takes precedence in being published, acessible and subsequently being cited [13]. It is essential that the avenues in digital dentistry that are being explored in under-developed or developing countries are also highlighted in a specific and focused bibliometric analysis, so that key areas of interest are identified, thereby highlighting domains where the margin of exploration exists, and research funding can be directed and prioritized. Authors and researchers working in digital dentistry can be identified for possible collaborations, ensuring that the revolutionary benefits which digital dentistry promises can have a wider reach in such geographical locations. This study was planned to determine how various research publications in different domains of digital dentistry conducted in low- and lower-middle income countries, classified according to the World Bank List [14] have competed for the number of citations, and impacted the development of scientific evidence and technology in digital dentistry. Therefore, the aim of this study was to conduct bibliometric and citation analysis of the top-cited articles from low- and lower-middle income countries, on the use and application of digital technology in dentistry. Various features of the studies were analyzed, including co-authorship analysis, co-occurrence of author keywords, bibliographic coupling and co-citation authorship analysis. In this way, avenues and areas in dentistry which have not been explored for the use of digitalization can be identified for the interest of the scientific community.

## Methodology

**Fig. 1:**
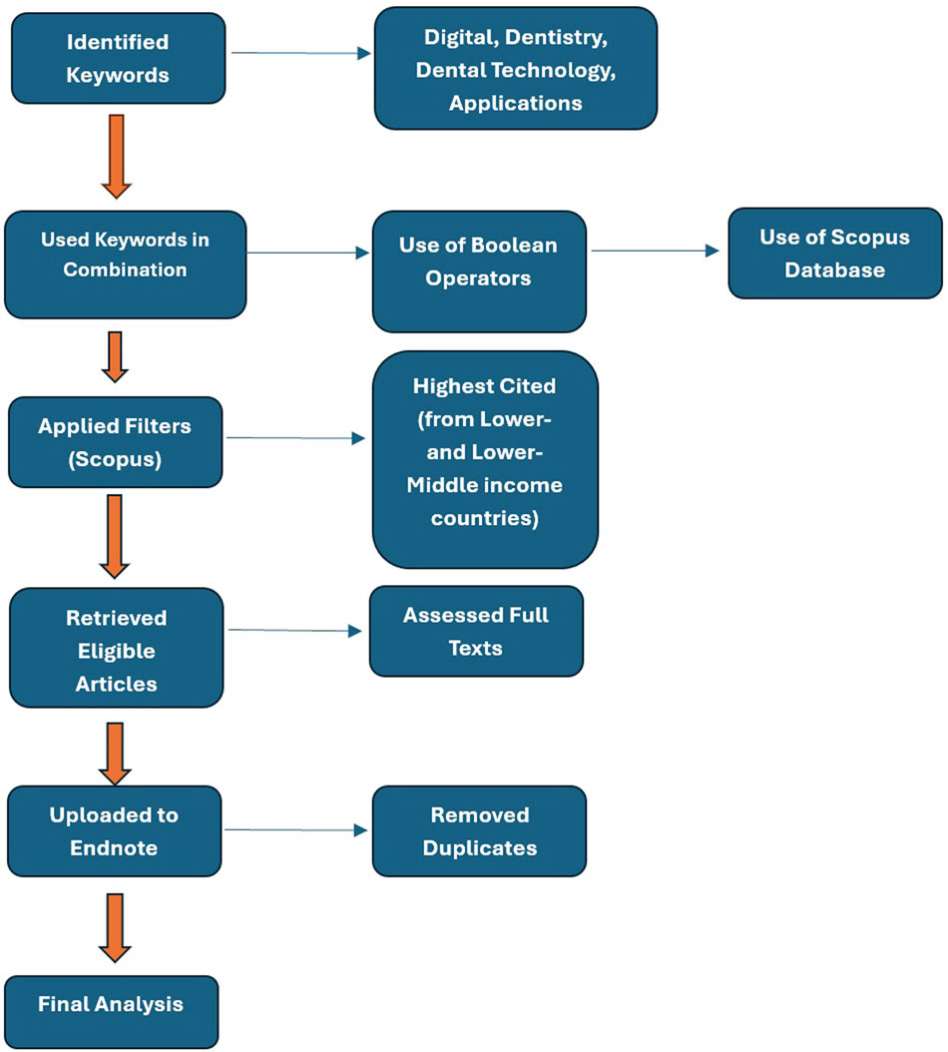
Flowchart illustrating the search and retrieval process of studies from lower- and lower-middle income countries using Scopus. Key words were identified, then used in various combinations in Scopus database, with application of filters for “Highest Cited” and “Country/Territory”, followed by retrieval of full-text articles, with selected citations uploaded to Endnote for analysis.

For this bibliometric analysis, the electronic search of relevant papers was conducted in October 2023. A search strategy was developed to identify articles on the Scopus database and retrieve records of peer-reviewed papers. The Scopus database was selected for analysis because of its accessibility and coverage of more research articles compared to other similar databases. The structured search strategy was formulated using different combinations of keywords including “Digital”, “Dentistry”, “Dental Technology”, “Applications”, “Digital Dentistry”, “Low Income Countries” and “Lower-Middle Income Countries” and similar terms, selecting the order of retrieval based on the highest number of citations. Further, by using Scopus filters of “Highest Cited” and “Country/Territory”, the relevant articles were retrieved using the name of individual countries placed in the categories of interest according to the World Bank List [14]. The initial list of papers, all from low- and lower-middle income countries, was uploaded to Endnote, and duplicates were removed. The flowchart for the search strategy is given in Fig. 1.

### Inclusion criteria

The eligibility of the retrieved studies was based on significant content related to the use of digital technology in dentistry, being full text, and available in the English language, with no restrictions applied to the publication date.

### Exclusion criteria

Any study with a citation count of less than ten was excluded.

The scrutiny of titles and abstracts during the initial search was performed by two reviewers (SA and AL). Any discrepancies were resolved by discussion with the third reviewer (FU). The target was to compile a list of articles based on the most cited publications relevant to Digital Dentistry. Once this was achieved, the data extracted from the selected articles was uploaded to SPSS version 23 for analysis. The information that was noted for each included publication comprised the title of the article, type of study, study design, journal and year of publication, author details, dental specialty in which digital technology was applied, country and institute where the study was conducted, network analysis based on co-citations, keywords, and the number of citations.

The study analyzed key thematic trends and research directions reflected in the included articles. The descriptive analysis, citation analysis, and mapping of the bibliometric data were performed using VOS software (version 1.6.20).

### Results

A total of 44 articles were selected for inclusion, published between the years 2009 and 2022 (Table 1).

**Table 1:** Publications included for the bibliometric analysis (*n* = 44).

S. no	Titles	Citations
1	Evaluation of the marginal fit of a zirconia ceramic computer-aided machined (CAM) crown system	144
2	3D and 4D printing in dentistry and maxillofacial surgery: Printing techniques, materials, and applications	133
3	Accuracy of ceramic restorations made with two CAD/CAM systems	105
4	Accuracy of three-dimensional dental resin models created by fused deposition modeling, stereolithography, and Polyjet prototype technologies: A comparative study	66
5	A survey on skills for cone beam computed tomography interpretation among endodontists for endodontic treatment procedure	58
6	Current status and applications of 3D scanning in dentistry	44
7	New evolution of cone-beam computed tomography in dentistry: Combining digital technologies	40
8	Visual and digital tooth shade selection methods, related effective factors and conditions, and their accuracy and precision: A literature review	38
9	Accuracy of an intraoral digital impression: A review	34
10	Digital Smile Design—An innovative tool in aesthetic dentistry	33
11	Digital implant impression technique accuracy: A systematic review	32
12	Wear resistance, color stability and displacement resistance of milled peek crowns compared to zirconia crowns under stimulated chewing and high-performance aging	26
13	A comparative evaluation of intraoral and extraoral digital impressions: An in vivo study	26
14	CAD/CAM-Assisted Auricular Prosthesis Fabrication for a Quick, Precise, and More Retentive Outcome: A Clinical Report	25
15	Comparison of dimensional accuracy of conventionally and digitally manufactured intracoronal restorations	25
16	Assessment of digital literacy and use of smart phones among Central Indian dental students	24
17	Flexible flow line scheduling considering machine eligibility in a digital dental laboratory	22
18	Influence of Preparation Type and Tooth Geometry on the Accuracy of Different Intraoral Scanners	21
19	Digitization and its futuristic approach in prosthodontics	20
20	Integrating digital technologies in dentistry to enhance the clinical success	19
21	A new digital approach for measuring dentin translucency in forensic age estimation	19
22	Novel Trends in Dental Color Match Using Different Shade Selection Methods: A Systematic Review and Meta-Analysis	18
23	Comparative evaluation of a novel smart-seal obturating system and its homogeneity of using cone beam computed tomography: In vitro simulated lateral canal study	18
24	Evaluation of the accuracy of orthodontic models prototyped with entry-level LCD-based 3D printers: a study using surface-based superimposition and deviation analysis	18
25	digital radiography in detecting approximal caries lesions in posterior permanent teeth: an in vivo study	17
26	Electro-optical system for the automated selection of dental implants according to their color matching	17
27	Significance of haptic and virtual reality simulation (VRS) in the dental education: A review of literature	17
28	Revolutionizing Restorative Dentistry: An Overview	16
29	A novel digital dentistry platform based on cloud manufacturing paradigm	16
30	Forensic dental age estimation by measuring root dentin translucency area using a new digital technique	14
31	Quantitative analysis of KTP laser photodynamic bleaching of tetracycline-discolored teeth	14
32	Primary evaluation of shape recovery of orthodontic aligners fabricated from shape memory polymer (A typodont study)	14
33	Maintenance of space by innovative three-dimensional-printed band and loop space maintainer	13
34	Digital dental photography	13
35	Digital imaging in dentistry: A review	13
36	Dental Caries early detection using Convolutional Neural Network for Tele dentistry	12
37	Expectation and reality of guided implant surgery protocol using computer-assisted static and dynamic navigation system at present scenario: Evidence-based literature review	12
38	Mobile dental photography: a simple technique for documentation and communication	11
39	Digital cytopathology	11
40	Evaluation of fully automated cephalometric measurements obtained from web-based artificial intelligence driven platform	10
41	Investigation of patient-specific maxillofacial implant prototype development by metal fused filament fabrication (Mf3) of ti-6al-4v	10
42	Automatic segmentation of lower jaw and mandibular bone in digital dental panoramic radiographs	10
43	Accuracy of different laboratory scanners for scanning of implant-supported full arch fixed prosthesis	10
44	Properties of CAD/CAM 3D Printing Dental Materials and Their Clinical Applications in Orthodontics: Where Are We Now?	10

## Bibliometric information of included articles

### Year of publication

From the articles that were first published in the year 2009, a constant surge was noted  in the number of articles published in each progressing year along with a rise in the number of citations. The highest number of articles were published in the year 2021 (*n* = 8), while six articles each were published in the years 2020, 2018, and 2017.

### Type of articles

Of the 44 articles, most of the studies were original articles (*n* = 23, 52.3%), followed by review articles (*n* = 16, 36.4%), as presented in Table 2.

**Table 2:** Distribution of articles according to study types (*n* = 44).

S.No	Types of articles	Frequency	Percentage
1	Original articles	23	52.3
2	Review (Literature, Scoping)	16	36.4
3	Systematic review and/or Meta-analysis	2	4.5
4	Case reports	1	2.3
5	Editorial/commentary	1	2.3
6	Conference proceeding	1	2.3

### Study design

The original articles included in our study (*n* = 23) were further categorized into whether the study design was in-vitro or in-vivo. Most of the articles were found to be in-vitro studies (40.9%).

### Country distribution

According to the country where the study was conducted or to which the first author belonged, about 54.5% (*n* = 24) of the studies were from India. The second highest number of articles were published from Iran (*n* = 7, 15.9%), followed by Egypt (*n* = 6, 13.6%). There were two articles each from Lebanon and Nepal, and one each from Pakistan, Syria, and Ukraine.

### Journal distribution

The articles included in the citation analysis were published in 33 journals, with The Journal of Indian Prosthodontic Society ranking first with publication of four relevant articles, followed by The Journal of Prosthetic Dentistry with three articles.

### Specialization

Most articles on digital dentistry were published in the domain of General Dentistry (*n* = 15, 34.1%), followed by Prosthodontics (*n* = 9, 20.5%), and Operative/Restorative Dentistry and Orthodontics with six articles each. There were four articles from Implantology, two from Oral Surgery, and one each from Endodontics and Periodontology.

## Co-authorship analysis

A co-authorship network analysis was performed keeping the authors and countries as units of analyses. The results revealed that there was no significant link strength between the authors as they were associated individually with each publication. The top authors in terms of citation counts were Baig M.R, Tan K.B and Nicholls J.I. (Complete information accessible in Supplementary Appendix 1).

**Fig. 2:**
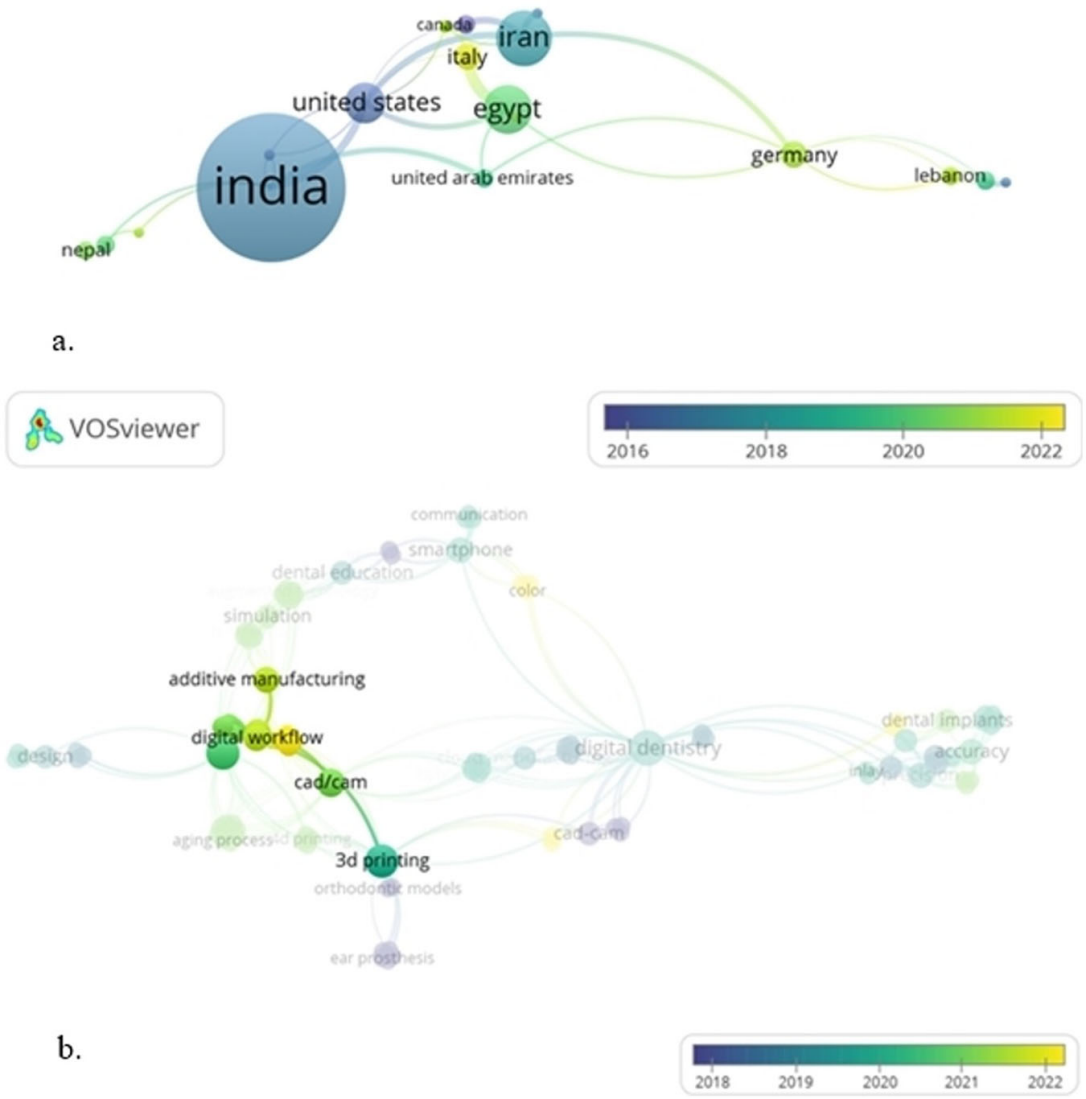
Overlay visualization of network analysis. **a** Country-wise co-authorship analysis. The node size represents the total number of articles published by the co-authors country-wise. Cluster consists of a group of closely related nodes carrying a particular color. **b** Contemporary areas of interest within the domain of digital dentistry. Large circles indicate more relevant and strongly related terms being positioned close to each other. The lines between terms indicate existent relationships with thicker lines representing a stronger link.

For country-wise co-authorship analysis, 24 countries were identified with the largest connected dataset. Of these 24 countries, India exhibited the highest link strength of 4.0 (total number of documents: 22, citations: 685), followed by the US (total number of documents: 5, citations: 420) and Iran (total number of documents: 7, citations: 283) Fig 2a.

## Co-occurrence of author keywords

The overlay visualization revealed that the highest occurring keywords were 3D printing (link strength 5.0), CAD/CAM (link strength 3.0), and cone beam computed tomography (link strength 2.0). Moreover, it was noted that in the recent era within the domain of digital dentistry, greater interest has been developed in digital workflow, CAD/CAM, and additive manufacturing processes in 3D printing (Fig. 2b).

## Citation analysis

A citation network analysis was performed keeping the sources and countries as units of analysis. The results demonstrated that the Journal of Prosthetic Dentistry had a considerable impact with the highest number of published documents (3) and with a citation count of 275, followed by others. However, no significant link strength was observed between these journal sources. The citation analysis trend for countries revealed that the majority of citations (685) were from India for a total of 22 documents, followed by the US (total documents: 5, citations: 420) and Iran (total documents: 7, citations: 283) (Supplementary Appendix 2).

## Bibliographic coupling

The bibliographic coupling for sources revealed that the Journal of Indian Prosthodontic Society had a significant impact with the highest link strength of 15.33 (total documents: 2, citations: 51), followed by the Journal of Prosthetic Dentistry (link strength: 8.0, total documents: 3, citations: 275) and Journal of Esthetic and Restorative Dentistry (link strength: 3.0, documents: 2, citations: 49).

## Co-citation authorship analysis

**Fig. 3:**
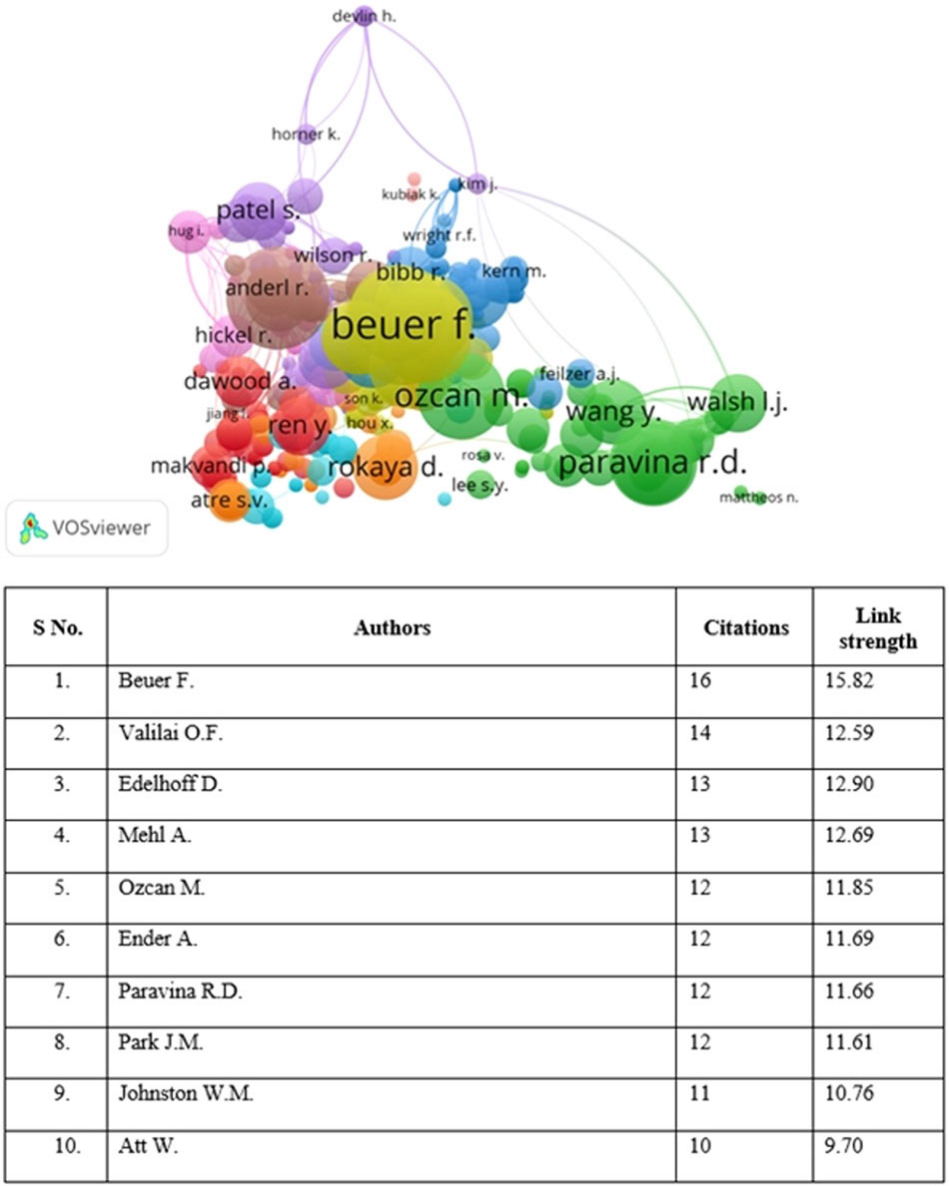
Overlay visualization of co-citation analysis for authors. A group of closely related nodes are represented via clusters carrying similar color. The node size represents the number of citations and link strength.

For co-citation authorship analysis, a total of 4720 authors were identified. However, most of these were not inter-connected, and hence the largest dataset of 978 connected authors was identified with the top ten authors in terms of exhibiting the highest link strength and citations (Fig. 3).

## Discussion

Bibliometric analyses hold substantial value in scientific research [3]. Through the systematic evaluation of publication trends, citation patterns, and authorship networks, researchers gain insights into the evolution of specific fields, such as Endodontics, Orthodontics, Prosthodontics, and Digital dentistry [15–18]. Therefore, bBibliometrics facilitates the identification of classic work, emerging trends, collaboration dynamics, and gaps in research.

Digital health encompasses healthcare and technology, employing digital tools and platforms to enhance the delivery of medical services. This approach leverages technologies like mobile apps, wearable devices, and telemedicine to monitor, diagnose, and manage health conditions [19]. Likewise, digital dentistry seamlessly incorporates cutting-edge technologies like electronic health records, practice management software, digital radiographs, Cone Beam Computed Tomography (CBCT), virtual reality, and advanced technologies such as CAD/CAM systems. This paradigm shift enhances precision, efficiency, and patient outcomes, marking an evolution in dentistry [20].

A recent bibliometric analysis of digital dentistry by Buddhikot et al. comprehensively evaluates the trends of publications within digital dentistry [12]. In this study, it was noted that the United States and Europe dominated the co-authorships and co-citations statistics, as well as citation counts [12]. It has been suggested that digital technologies can bring an improvement in health delivery and outcomes, as well as reduction of medical spending in low-resource settings [21, 22]. Contrarily, the swift progression of digital health technologies in some geographical locations as compared to others has resulted in disparities in healthcare systems [22]. With this context, in order to better understand the publication landscape, the authors decided to conduct this bibliometric analysis with a focus on publications from developing and under-developed countries, classified as low- and lower middle-income countries by the World Bank.

We evaluated a total of 44 articles, aligning with our search strategy and inclusion/exclusion criteria, which specifically focused on these countries. Diverging from recent studies in this domain, our approach encompassed both open-access and subscription-based publications [19]. Notably, an upward trend in publications emerged from 2015 onwards, with a particular upsurge since 2019–2022. The prevalent index keywords included 3D printing, CAD/CAM, and Cone Beam Computed Tomography (CBCT). These findings are consistent with other publications [12]. It is interesting to note that although 3D printing is considered significantly costly for it to be regarded as a routine process even in every developed country, yet the growing attention this procedure is gaining in publications from low- and lower-middle income countries indicates that researchers in developing countries are predicting its projected applicability and utility in their contexts, based on the inherent benefits of customization and accuracy that it promises [23]. In addition, more recently, a growing focus in digital workflow is also evident, with similar benefits of streamlined and faster production expected [9]. Therefore, this provides reason to believe that analysis of citations highlights impactful scientific evidence, which can then dictate clinical procedures, protocols and aid in informed decision making. In this way, through the information provided by this bibliometric and citation analysis, new clinical recommendations can be developed for utilization of 3D printing and other digital technology in clinical setups.

Through our results, it was noted that India had the most number of publications. In addition, India also had the highest co-authorship, co-citations, and citations trends. This could be an indication of multiple meaningful collaborations taking place amongst and with the researchers from this country, who are working on different aspects of digital dentistry. In contrast, Pakistan being one of the most populous countries in the region, had only one publication which could be included. The study from Pakistan was a literature review which focused on the use of digital technology in the form of haptic and virtual reality (VR) for education of dental students [24].

The Journal of Indian Prosthodontic Society emerged as the leading contributor in terms of publications, closely followed by the Journal of Prosthetic Dentistry. This notable pattern is illustrated through bibliographic coupling, highlighting a significant connection between the two journals. This correlation aligns seamlessly with the thematic focus, as keywords such as 3D printing, CAD/CAM, and CBCT, particularly in the domain of implant dentistry, are commonly associated with the field of Prosthetic dentistry.

Among the publications incorporated in our study, only 52% constituted original articles. This highlights the need for increased efforts in conducting original research within low- and lower-middle income countries. Such endeavors are crucial to fully unlock the potential of digital technologies in advancing dental care within these regions. We deliberately excluded artificial intelligence (AI) from our analysis due to the prevalent observation that many AI tools are not yet widely deployed in practical, real-world scenarios. Our decision was driven by the intention to specifically visualize the impact of research that has direct relevance and applicability in real-world contexts. By focusing on existing, implemented technologies rather than speculative or experimental AI applications, our analysis seeks to provide a more accurate representation of the current influence of digital dentistry research in practical, everyday settings.

Whilst this bibliometric analysis presents valuable insights into the domain of digital dentistry, it is imperative to acknowledge several limitations inherent in this evaluation. The choice of Scopus as the sole database may introduce selection bias, potentially excluding relevant publications not indexed in this database. The decision to include only English-language papers may lead to the oversight of valuable contributions in other languages. Setting the citation count at 10 or above for inclusion might exclude potentially relevant studies with lower citation counts but with significant contributions or those with unique topics having the potential to gain significant citations in the future. Hence, the currently used threshold may overlook emerging research areas. Nonetheless, this cut-off was selected with the anticipation of a higher chance of observing meaningful associations between different parameters related to the citations of the included articles. The subjectivity inherent in the review process, despite involving multiple reviewers, also introduces a degree of interpretation that may influence article selection.

Processing and analyzing this bibliometric data using VOS software was resource-intensive. However, the resulting visualization of the relationships between publications and authors gives this research a more nuanced understanding of the collaborative networks and influential contributors in the field of digital dentistry, which can be considered an additional strength of this citation analysis.

## Future recommendations and research

Moving forward, there is a pressing need to prioritize original research initiatives within low- and lower-middle income countries to foster the development of innovative digital dental solutions tailored to local contexts. This entails supporting scientific efforts in these regions and advocating for more inclusivity in academic publishing to ensure that less represented scientific work gets noticed. Additionally, efforts should be directed toward overcoming practical challenges associated with the adoption of digital dental technologies, including addressing issues of cost-effectiveness, infrastructural limitations, and workforce capacity.

## Conclusion

This bibliometric analysis provides insight into the field of digital dentistry based on citation counts, with a specific focus on low- and lower-middle income countries, identifying India in prominently leading the way as a significant contributor to this evolving landscape. Our analysis uncovers key thematic trends, emphasizing the crucial role of technologies like 3D printing, CAD/CAM, and CBCT in digital dentistry. The study underscores the imperative for increased original research efforts within these regions to fully harness the potential of digital technologies, and prevent the disparity in the representation of research endeavors.

### Supplementary information

Supplementary Information

## Data Availability

All the data used are available within the manuscript.
