# Supplementary material for: A bibliometric analysis of scientific literature in digital dentistry from low- and lower-middle income countries
Source: BDJ Open. 2024 May 25;10:38. doi: 10.1038/s41405-024-00225-4 (PMC11127973; doi:10.1038/s41405-024-00225-4)

Appendix 1. Authorship and Co-authorship analysis

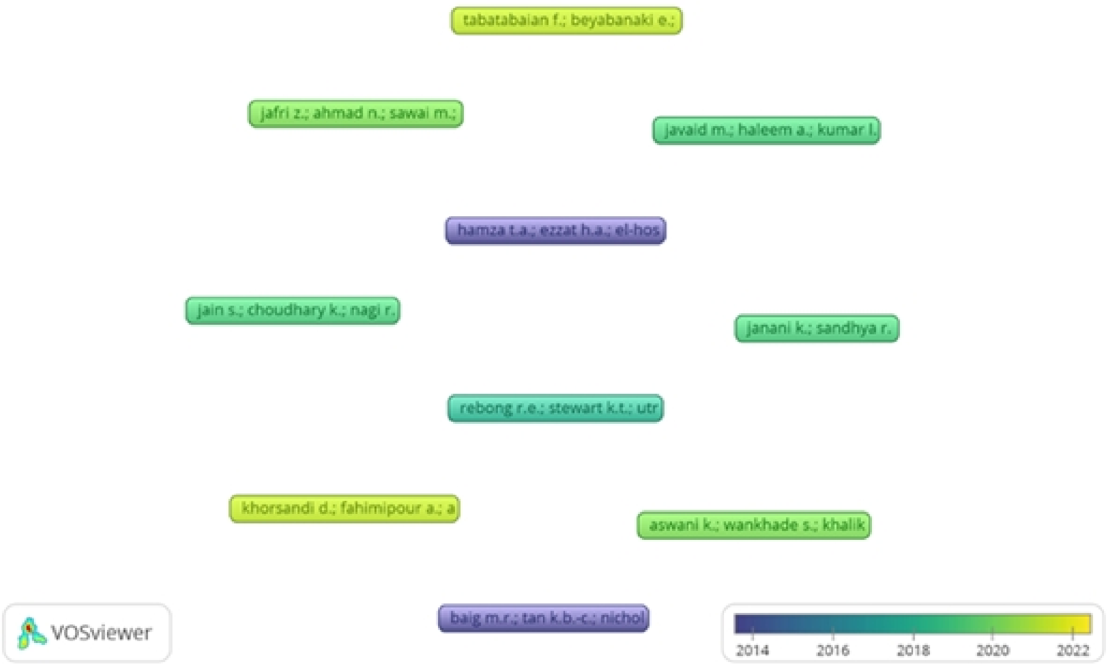

S No.	Authors	Citations
1.	Baig M.R.; Tan K.B; Nicholls J.I	145
2.	Khorsandi D.; Fahimipour A.; Abasian P.; Saber S.S.; Seyedi M.; Ghanavati S.; Ahmad A.; De Stephanis A.A.; Taghavinezhaddilami F.; Leonova A.; Mohammadinejad R.; Shabani M.; Mazzolai B.; Mattoli V.; Tay F.R.; Makvandi P.	135
3.	Hamza T.A.; Ezzat H.A.; El-hossary M.M.K.; El Megid Katamish H.A.; Shokry T.E.; Rosenstiel S.F.	105
4.	Rebong R.E.; Stewart K.T.; Utreja A.; Ghoneima A.A.	66
5.	Janani K.; Sandhya R.	58
6.	Javaid M.; Haleem A.; Kumar I.	44
7.	Jain S.; Choudhary K.; Nagi R.; Shukla S.; Kaur N.; Grover D.	41
8.	Tabatabaian F.; Beyabanaki E.; Alirezaei P.; Epakchi S.	39
9.	Jafri Z.; Ahmad N.; Sawai M.; Sultan N.; Bhardwaj A.	36
10.	Aswani K.; Wankhade S.; Khalikar A.; Deogade S.	35

Appendix 2. Overlay visualization of co-citation analysis for countries

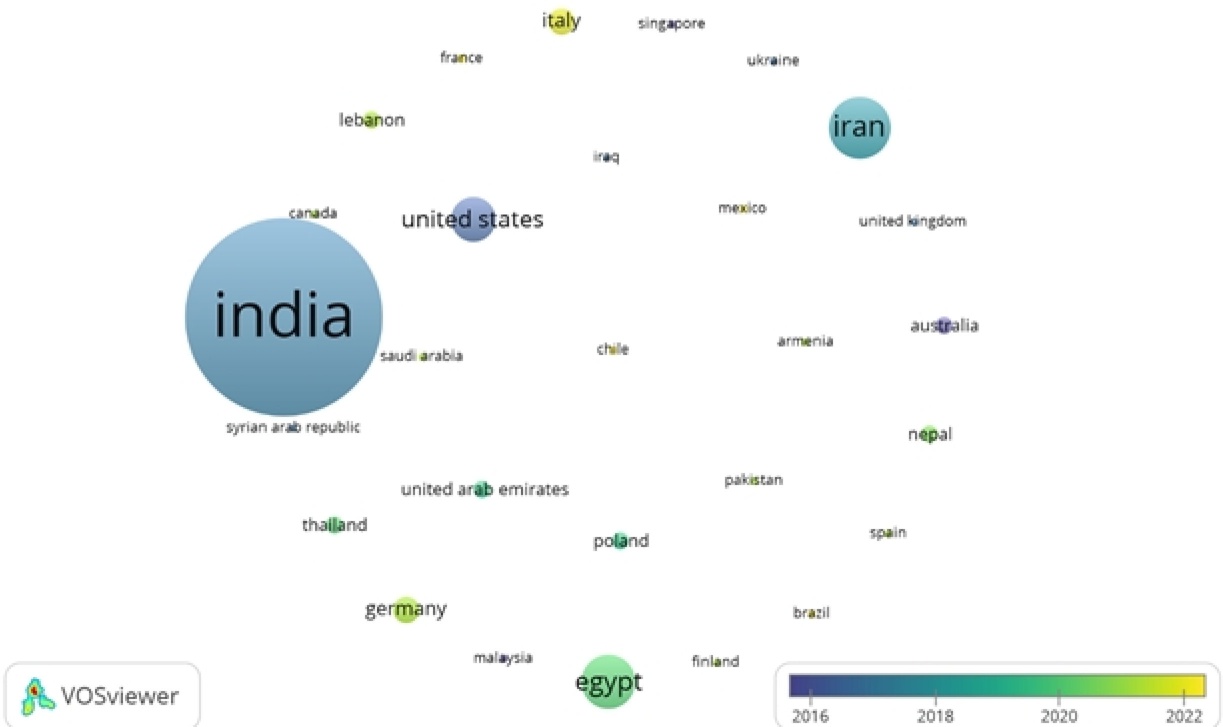

Supplement: Supplementary file 1 — Supplementary Information [file 41405_2024_225_MOESM1_ESM.pdf]
